# Diagnosing Diabetic Retinopathy With Artificial Intelligence: What Information Should Be Included to Ensure Ethical Informed Consent?

**DOI:** 10.3389/fmed.2021.695217

**Published:** 2021-07-21

**Authors:** Frank Ursin, Cristian Timmermann, Marcin Orzechowski, Florian Steger

**Affiliations:** Institute of the History, Philosophy and Ethics of Medicine, Ulm University, Ulm, Germany

**Keywords:** informed consent, information process, machine learning, diabetic retinopathy, ethics

## Abstract

**Purpose:** The method of diagnosing diabetic retinopathy (DR) through artificial intelligence (AI)-based systems has been commercially available since 2018. This introduces new ethical challenges with regard to obtaining informed consent from patients. The purpose of this work is to develop a checklist of items to be disclosed when diagnosing DR with AI systems in a primary care setting.

**Methods:** Two systematic literature searches were conducted in PubMed and Web of Science databases: a narrow search focusing on DR and a broad search on general issues of AI-based diagnosis. An ethics content analysis was conducted inductively to extract two features of included publications: (1) novel information content for AI-aided diagnosis and (2) the ethical justification for its disclosure.

**Results:** The narrow search yielded *n* = 537 records of which *n* = 4 met the inclusion criteria. The information process was scarcely addressed for primary care setting. The broad search yielded *n* = 60 records of which *n* = 11 were included. In total, eight novel elements were identified to be included in the information process for ethical reasons, all of which stem from the technical specifics of medical AI.

**Conclusions:** Implications for the general practitioner are two-fold: First, doctors need to be better informed about the ethical implications of novel technologies and must understand them to properly inform patients. Second, patient's overconfidence or fears can be countered by communicating the risks, limitations, and potential benefits of diagnostic AI systems. If patients accept and are aware of the limitations of AI-aided diagnosis, they increase their chances of being diagnosed and treated in time.

## Introduction

The timely referral of patients with diabetic retinopathy (DR) to ophthalmic examination is crucial to avoid visual impairment and blindness (1). Artificial intelligence (AI)-aided diagnosis has been developed to speed up access to an ophthalmology specialist and to reduce healthcare costs (1). Although the idea of automated DR screening stems from the late 1990s, the major advances in computing power in the early 2010s made it possible to handle the heavy load of retinal images (2, 3). The diagnosis of DR through AI systems is commercially available for primary care settings since 2018 (4, 5). The system, called IDx-DR, runs on a retinal camera (Topcon NW400) and analyzes images of the eye utilizing an AI algorithm (6). The system's output is a recommendation either to refer patients to an eye care professional when it identifies more than mild DR or to suggest rescreening in 12 months. By this, it is the first device that provides an autonomous screening decision, receiving market approval by the US Food and Drug Administration (FDA) (5). For ethics, a novel and unique feature of this AI-aided device requires a critical assessment: the IDx-DR intentionally generates an autonomous recommendation that actually is a diagnosis without needing the oversight of a physician, although physicians are normally responsible for diagnosis.

This situation engenders a certain urgency to clarify the ethical issues of informed consent because the device is already commercially available. Hitherto, “what to tell the patient” is discussed in terms of legal analyses for the US and the EU (7–9). However, a specific answer to the ethical question “what information should be included in the information process if AI is involved in the diagnosis” has not been provided regarding the new devices for diagnosing DR. The ethical challenges stem from the opacity of black-box algorithms, potential biases within the training data, tensions between improving healthcare and generating profit, and the liability in case of performance errors (10).

To mitigate these ethical challenges, it has been tentatively proposed to educate physicians about the construction of AI systems, their training data and limitations, and to create ethical guidelines that go beyond legal requirements (11). These measures are meant to improve the information process and foster patients' trust in AI systems. However, it remains unclear what specific information should be included in the information process for diagnosing DR with AI systems in a primary care setting. While commentators question whether the involvement of diagnostic AI must be disclosed on legal grounds at all (7), the authors of this article believe that an omission of this fact is unethical as it may amount to a form of deceit. The aim of the article is to develop a checklist to ethically safeguard the informed consent process. We take the IDx-DR system as an example for other commercially available AI-aided tools for diagnosing DR, such as EyeArt that received FDA approval in June 2020 (12–14).

## Materials and Methods

The research question is as follows: What new information should be included in the information process to ethically secure informed consent when AI is used for DR diagnosis? To answer this question, we developed a list of contents and their ethical justification based on a systematic literature search. Two distinct searches were conducted. The first search aimed specifically at the question of AI-aided DR diagnosis. Because this initial search yielded no significant results, a second broader search was conducted on AI-aided diagnosis in general.

Because the topic is new and unique, we decided to apply an explorative method of coding and pooling the contents derived from the literature. Accordingly, an ethics content analysis was conducted inductively to extract two features of included publications: (1) novel information content for AI-aided diagnosis and (2) the ethical justification for its disclosure within the information process. The results from this second search allowed to formulate specific novel content that should be included into the information process for AI-aided DR diagnosis. The criterion for novelty was that a content followed from applying AI and not merely from the diagnostic procedure. We related these results to the five aspects of informed consent: (1) information disclosure to safeguard autonomous decisions, (2) the patient's capacity to understand the information, (3) voluntariness of the decision, (4) the competence to make decisions, and (5) the decision itself (15). We will focus on the aspects of informed consent to which our findings relate (aspects 1–3).

Literature was searched in PubMed and Web of Knowledge (Web of Science Core Collection) databases on January 14, 2021. Search string 1 was {[(consent) OR (information)] AND [(artificial intelligence) OR (ai) OR (machine learning) OR (deep learning) OR (algorithm)] AND (diabetic retinopathy)}. This first search yielded 537 records. After removing duplicates, titles, and abstracts of *n* = 401 records were screened. Publications were excluded that do not address ophthalmological diagnosis. Full text was assessed of *n* = 53 publications and screened for “consent,” “inform^*^,” and “patient.” Only *n* = 4 articles addressed in which setting and how consent was obtained for AI-aided DR diagnosis. No publication specified the content of information disclosure for informed consent.

Due to the lack of specific content on information disclosure for AI-aided diagnosing of DR, a broader search was conducted. Search string 2 was [(Informed Consent) AND (artificial intelligence)] for PubMed and Web of Knowledge. This search yielded 60 records. After removing duplicates, titles and abstracts of *n* = 43 records were screened. Publications were excluded that do not address medical AI. Full text was assessed of *n* = 24 publications. In this step, publications were excluded, which addressed decision making in surgery (*n* = 4); direct-to-consumer psychotherapy apps (*n* = 1); digital consent in public health research (*n* = 2); digital consent more broadly (*n* = 2); medical education (*n* = 1); robots and self-driving cars (*n* = 1); consent to mobile neuroimaging (*n* = 1); and intelligent assistive technology in elderly care (*n* = 1). In total, *n* = 11 articles met the study's objective. The inclusion criterion was the mention of novel content to be included in the information process when AI is used for diagnosis.

## Results

### Narrow Search on Artificial Intelligence-Aided Diabetic Retinopathy Diagnosis

All publications whose full text was assessed reported research in AI-aided methods of DR diagnosis. References to the use of consent forms were not found with database searching. There was no article that reported specific content of information for obtaining informed consent in patient care. However, two articles reported research in primary care settings that is ethically relevant for the assessment of IDx-DR. The devices reported in both studies were utilized by minimally trained healthcare workers, not by a physician (16, 17). This is also the case with IDx-DR. The unique feature of the study by Natarajan et al. is that they utilized an offline AI software with a smartphone-based fundus camera (17). IDx-DR needs an internet connection to the company's servers and a non-mydriatic fundus camera, which creates a dependency and is more expensive than offline systems.

### Broad Search on Artificial Intelligence-Aided Diagnosis

In total, eight novel contents that should be included in the information process on ethical grounds are described in the general literature on AI-aided diagnosis. These contents are mainly concerned with the technical peculiarities of machine learning (ML) algorithms. Table 1 charts these contents accompanied by the given ethical justifications for disclosing them within the information process. Within the ethical framework of informed consent, five items belong to the aspect of information disclosure, two items foster understanding of the information, and one item secures voluntariness. The voluntariness can be secured by providing an adequate alternative to AI-aided testing, i.e., direct referral to ophthalmic examination, but this would undermine the expected positive effects of autonomous diagnosis. Understanding can be fostered by describing the AI's input and output data as well as explaining the AI's training and how it generates its output by learning from examples. The information to be disclosed contain the three risks of algorithmic mismatch, algorithmic bias, and cyber-attack. Furthermore, it should be disclosed whether and how patients' data are processed.

**Table 1:** Novel information for artificial intelligence (AI)-aided diagnosis and the ethical justification of its inclusion in the information process.

**Novel information for AI-aided diagnosis**	**Ethical justification of its inclusion in the information process**	**Source**
(i) Disclosure of algorithmic decision support without the oversight of a physician	Transparency toward devices and technology respects the autonomy, values, and preferences of patients	(7, 9)
(ii) Description of the AI's input and output data	Because most AI/machine learning (ML) algorithms are black boxes, at least the input and output data should be described to understand the nature of the procedure	(10, 18, 19)
(iii) Explanation of the AI's training and how it generates its output by learning from examples	Patients have the right to know how the AI algorithm reaches its decisions if it generates medical decisions without any meaningful intervention from a physician	(10, 18, 19)
(iv) Disclosure of the risk of cyber-attack	Patients should be aware of the new risks that emerge from the technical peculiarities of AI/ML algorithms due to some degree of predictive uncertainty	(20)
(v) Disclosure of the risk of algorithmic bias	idem	(20)
(vi) Disclosure of the risk of algorithmic mismatch (false-positive or false-negative results)	idem	(20)
(vii) The right to a second opinion by a trained physician	According to the informed consent paradigm, there is not only a negative right to refuse certain interventions but also a positive right to be provided with alternative diagnostics or treatments performed by a physician	(8, 19, 21)
(viii) Disclosure of whether and how the patient's data will be used beyond the diagnosis	Data ownership, security, and privacy demands disclosure of what kind of information is obtained and how and if it will be accessed, linked, and used	(22)

The most controversial item is the disclosure of algorithmic decision support without the oversight of a physician. At present, there is no ethical and legal consensus whether disclosing the application of an opaque medical AI is required for informed consent in the USA and the EU (18). A legal obligation to disclose the utilization of medical AI may not exist according to the current legal doctrine in the USA (7). However, if patients specifically asks whether or not medical AI is involved in diagnosis, physicians may have to disclose its application (7). In the EU, fully automated decision making without human involvement is prohibited, and therefore, physicians remain responsible for medical decisions including diagnosis (General Data Protection Regulation, GDPR, Article 22) (9, 23). However, this still allows AI-aided diagnosis.

## Discussion

**Figure 1:**
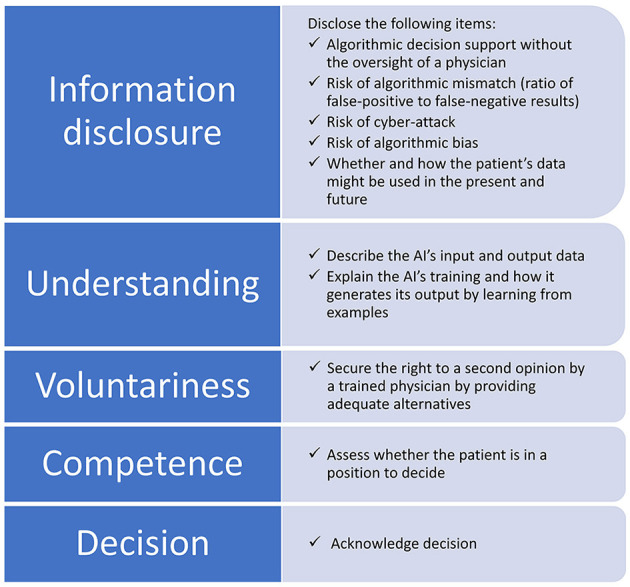
Items to be disclosed within the information process for obtaining informed consent when AI is involved in the diagnosis. The eight novel items are assigned to the aspects of information disclosure, understanding, and voluntariness, while competence and the decision itself remain untouched.

We discuss the eight identified items within the framework of informed consent. Figure 1 situates these items within the information process. Thereby, we provide a checklist to ethically safeguard internationally acknowledged standards for obtaining informed consent. We assigned the eight novel items to the first three aspects leaving aside the competence to make decisions and the decision itself because the new items do not interfere with them. Making sure patients understand the information and respecting their decision are important for all diagnoses, and they are not unique to AI-aided systems. To indicate the items' significance and ethical implications within different regulatory environments, we compare the situation in the USA and EU with regard to voluntariness and the right to a second opinion. By the example of these two issues, one can observe distinctly different ethical implications.

### Information Disclosure for Artificial Intelligence-Aided Diagnosis

The ethical and legal debate about “what to tell the patient” when medical AI is involved has just begun (7). The legal debate concentrates on issues of data protection and liability. As we propose, in addition to the usual items, the information process should include our eight-item list of novel information. This list, when complemented with basic patient-tailored descriptions, assists in informing about the complexity of AI processes from an ethical perspective.

The risk of algorithmic mismatch (ratio of false-positive to false-negative results) can be disclosed by showing how good an AI system performs, e.g., the IDx-DR has 87.2% sensitivity and 90.7% specificity (6). Patients should be aware that AI/ML algorithms have limited accuracy, but this also applies to doctors. An ethical issue could arise from the dependence on an internet connection that bounds the user to the offering company and is more expensive than offline systems due to the acquisition costs of the fundus camera. This may limit access to healthcare and is, therefore, a concern for justice.

The risk of algorithmic bias is more difficult to communicate. It is important to note that the IDx-DR system was trained on a diverse population of 819 participants and showed robust diagnostic accuracy regarding sex, race, ethnicity, lens status, and metabolic control (6). However, it has been observed that algorithms may include a racial bias due to the fact that their training datasets are not representative (24). Many developers of AI algorithms for detecting DR use the images from datasets like Kaggle, Messidor, or Eyepacs, which are not representative for human diversity (24). There is the chance of overfitting, which occurs when the underlying datasets are too homogenous and, therefore, prone to generalization problems (25). Therefore, AI systems may not perform well on diverse populations and increase the risk of misdiagnosis. Patients should be informed about possible biases in the training dataset and how they could affect the results of the DR examination. This could unintentionally end up in the (self-)exclusion of certain patient populations from access to AI-aided diagnostic systems due to the peculiarities of the data that has been used to train them.

### Improving the Understanding of Information

In standard circumstances, the duty to comprehensively inform patients requires physicians also to check whether a patient understands the nature of the procedure, its risks, and alternatives (18). As a basis, all this also holds true for AI-aided DR diagnosis. However, for informed consent to be comprehensive and understandable, we need to consider the following different types of explanations to also build trust in medical AI: why- and how-explanations. Why-explanations are explanations for confidence that honor the principle of transparency. For example, doctors can inform that the FDA has approved the IDx-DR system as “Software as Medical Device” in 2018 and, therefore, patients should be confident in its safe and effective use. By referring to a trusted agency, it is assumed that patients will associate the approved technology with the same level of trustworthiness.

How-explanations are explanations for trust that honor the principle of justification. For example, doctors can inform that IDx-DR is a medical AI system that was trained to detect disease patterns in images using ML (26). To facilitate trust, the description of how such systems work is simplified, by unraveling how some of the easier-to-understand subprocesses within the larger system function. Due to technical peculiarities, there are ethical challenges if the patient insists on an extensive explanation regarding AI-aided diagnosis. This is because the patient asks for something the physician cannot answer to satisfactorily like in the case of an X-ray image. If the patient asks a radiologist why he has diagnosed a certain condition, the radiologist can point to an area on an X-ray image and, e.g., qualify certain pixels as an aberration from normality or as a space-consuming injury; in case of IDx-DR, the physician cannot.

Although the “black box” character of ML systems is an epistemic hurdle for explainability, it is not for diagnosis itself (2, 27). The more opaque an ML algorithm is, the more accurately it performs (28). There are attempts to overcome this epistemic hurdle by pointing to the pictorial contents that contribute to the algorithm's decision (29). This enables to interpret the output of AI-based diagnostic systems and to generate clinically meaningful results (30), but as long as these techniques are not readily available, the nature and architecture of ML algorithms must be sufficiently disclosed whenever the patient is eager to learn this information.

Insufficient comprehension of patient information conveyed through standard procedures has been observed (31). Therefore, innovative methods for improving patient comprehension of information provided in AI-aided DR diagnosis should be included. Best results can be achieved with complementing the information given with audiovisual content, e.g., video recordings; interactive digital materials, e.g., applications requiring active patient participation; or through teach–feedback process. All these methods significantly improve patient information compared with the standard form of patient information (32).

### Voluntariness and the Right to a Second Opinion

The great advantage of ML systems is that their algorithms improve when they are applied because they can continuously learn from new examples. This learning phase traditionally is considered to belong to research. Now, research and clinical application blend into each other. However, research and patient care have to be separated also in the case a continuously learning AI can be improved by more data (33). Usually, the FDA only approves “locked” AI systems that are not continuously learning and have to be reevaluated if new training data is used. This is an important information for patients who fear ending up as research subjects during routine examinations.

While regulators like the FDA in the USA and the Medical Device Regulation in the EU do address safety and effectiveness of “Software as Medical Device,” they do not address informed consent (34, 35). In the EU, Article 22 of the General Data Protection Regulation (GDPR) states that data subjects have the right to a second opinion and to human oversight if fully automated decision making takes place (23). Therefore, physicians remain responsible for medical decisions toward patients (9). The right to know how a medical diagnosis is made is backed by Article 13 of the GDPR, which states that an individual has the right to know “meaningful information about the logic involved” in processes that directly affect them (23). This holds true both for research and clinical settings. If translational research takes place, it must meet the ethical and legal requirements applying to research (cf., among others, the Declaration of Helsinki) (9).

## Conclusions

In total, eight novel contents should be included to obtain informed consent for AI-aided DR diagnosis for ethical reasons. Five items belong to the aspect of information disclosure, i.e., disclosing algorithmic decision support without the oversight of a physician, whether and how patients' data are processed as well as the three risks of algorithmic mismatch, algorithmic bias, and cyber-attack. Two items foster understanding of the information, i.e., describing the AI's input and output data as well as explaining the AI's training and how it generates its output by learning from examples. Voluntariness can be secured by disclosing the right to a second opinion by a trained physician, i.e., direct referral to ophthalmic examination, but this would undermine the expected positive value of the autonomous diagnosis.

Implications for the general practitioner are two-fold: First, if the physician's task is to inform the patient about the involvement of medical AI, then he or she must have the relevant knowledge and skills to make sure patients understand the implications of the use of such technology. It has been demanded to develop tailored training for physicians in the area of medical AI (18). We agree because the items we identified to be included in the information process require specialized knowledge. Specifically, physicians need to know how AI/ML applications are constructed, which data were used to train them, and what their limitations are (10).

The second implication for the general practitioner is that the patient's fears or overconfidence in AI-aided diagnosis must be addressed. This can be achieved by describing the risks and potential benefits of the AI system, e.g., by providing studies that compare diagnostic accuracy of medical AI compared with human eye doctors (10). If patients accept and are aware of the risks and limitations of AI-aided diagnosis, they will save a long wait for an ophthalmologist appointment.

## Data Availability Statement

The original contributions presented in the study are included in the article/supplementary material, and further inquiries can be directed to the corresponding author/s.

## Author Contributions

FU, CT, MO, and FS conceptualized the study, performed the data analysis, wrote, reviewed, and edited the article. FU wrote and prepared the original draft. All authors contributed to the article and approved the submitted version.

## Conflict of Interest

The authors declare that the research was conducted in the absence of any commercial or financial relationships that could be construed as a potential conflict of interest.

## References

[1] Vujosevic S Aldington SJ Silva P Hernández C Scanlon P Peto T. Screening for diabetic retinopathy: new perspectives and challenges. Lancet Diabetes Endocrinol. (2020) 8:337–47. 10.1016/S2213-8587(19)30411-5 32113513

[2] Ting DSW Peng L Varadarajan AV Keane PA Burlina PM Chiang MF. Deep learning in ophthalmology: the technical and clinical considerations. Progr Retinal Eye Res. (2019) 72:100759. 10.1016/j.preteyeres.2019.04.003 31048019

[3] Gardner GG Keating D Williamson TH Elliott AT. Automatic detection of diabetic retinopathy using an artificial neural network: a screening tool. Br J Ophthalmol. (1996) 80:940–4. 10.1136/bjo.80.11.940 8976718 PMC505667

[4] Savoy M. IDx-DR for diabetic retinopathy screening. Am Family Phys. (2020) 101:307–8. 32109029

[5] U.S. Food and Drug Administration. FDA Permits Marketing of Artificial Intelligence-Based Device to Detect Certain Diabetes-Related Eye Problems. (2018). Available online at: https://www.fda.gov/news-events/press-announcements/fda-permits-marketing-artificial-intelligence-based-device-detect-certain-diabetes-related-eye (accessed January 21, 2021).

[6] Abràmoff MD Lavin PT Birch M Shah N Folk JC. Pivotal trial of an autonomous AI-based diagnostic system for detection of diabetic retinopathy in primary care offices. NPJ Digit Med. (2018) 1:39. 10.1038/s41746-018-0040-6 31304320 PMC6550188

[7] Cohen IG. Informed consent and medical artificial intelligence: what to tell the patient? Georgetown Law J. (2020) 108:1425–1469. 10.2139/ssrn.3529576

[8] Miguel I de Sanz B Lazcoz G. Machine learning in the EU health care context: exploring the ethical, legal and social issues. Inform Commun Soc. (2020) 23:1139–53. 10.1080/1369118X.2020.1719185

[9] Mitchell C Ploem C. Legal challenges for the implementation of advanced clinical digital decision support systems in Europe. J Clin Transl Res. (2018) 3(Suppl. 3):424–30. 10.18053/jctres.03.2017S3.005 30873491 PMC6412598

[10] Schiff D Borenstein J. How should clinicians communicate with patients about the roles of artificially intelligent team members? AMA J Ethics. (2019) 21:E138–45. 10.1001/amajethics.2019.138 30794123

[11] Char DS Shah NH Magnus D. Implementing machine learning in health care - addressing ethical challenges. N Engl J Med. (2018) 378:981–3. 10.1056/NEJMp1714229 29539284 PMC5962261

[12] Benjamens S Dhunnoo P Meskó B. The state of artificial intelligence-based FDA-approved medical devices and algorithms: an online database. NPJ Digit Med. (2020) 3:118. 10.1038/s41746-020-00324-0 32984550 PMC7486909

[13] The Medical Futurist. FDA-Approved A.I.-Based Algorithms. Available online at: https://medicalfuturist.com/fda-approved-ai-based-algorithms/ (accessed June 8, 2021).

[14] Grzybowski A Brona P Lim G Ruamviboonsuk P Tan GS Abramoff M. Artificial intelligence for diabetic retinopathy screening: a review. Eye. (2020) 34:451–60. 10.1038/s41433-019-0566-0 31488886 PMC7055592

[15] Faden RR Beauchamp TL King NMP. A History and Theory of Informed Consent. New York, NY: Oxford University Press (1986). 11621442

[16] Kanagasingam Y Xiao D Vignarajan J Preetham A Tay-Kearney ML Mehrotra A. Evaluation of artificial intelligence-based grading of diabetic retinopathy in primary care. JAMA Network Open. (2018) 1:e182665. 10.1001/jamanetworkopen.2018.2665 30646178 PMC6324474

[17] Natarajan S Jain A Krishnan R Rogye A Sivaprasad S. Diagnostic accuracy of community-based diabetic retinopathy screening with an offline artificial intelligence system on a smartphone. JAMA Ophthalmol. (2019) 137:1182–8. 10.1001/jamaophthalmol.2019.2923 31393538 PMC6692680

[18] Amann J Blasimme A Vayena E Frey D Madai VI. Explainability for artificial intelligence in healthcare: a multidisciplinary perspective. BMC Med Inform Decis Mak. (2020) 20:310. 10.1186/s12911-020-01332-6 33256715 PMC7706019

[19] Broeckx N Lemmens C. Artificial or human intelligence: who is to blame? J Belgian Soc Radiol. (2018) 102:21. 10.5334/jbsr.1637

[20] Kiener M. Artificial intelligence in medicine and the disclosure of risks. AI Soc. (2020) 1–9. 10.1007/s00146-020-01085-w. [Epub ahead of print]. 33110296 PMC7580986

[21] Ploug T Holm S. The right to refuse diagnostics and treatment planning by artificial intelligence. Med Health Care Philos. (2020) 23:107–14. 10.1007/s11019-019-09912-8 31359302

[22] Manrique de Lara A Peláez-Ballestas I. Big data and data processing in rheumatology: bioethical perspectives. Clin Rheumatol. (2020) 39:1007–14. 10.1007/s10067-020-04969-w 32062767

[23] European Parliament, European Council. General Data Protection Regulation. (2016). Available online at: https://eur-lex.europa.eu/legal-content/EN/TXT/PDF/?uri=CELEX:32016R0679&from=EN (accessed November 5, 2020).

[24] Raman R Srinivasan S Virmani S Sivaprasad S Rao C Rajalakshmi R. Fundus photograph-based deep learning algorithms in detecting diabetic retinopathy. Eye. (2019) 33:97–109. 10.1038/s41433-018-0269-y 30401899 PMC6328553

[25] Sarhan MH Nasseri MA Zapp D Maier M Lohmann CP Navab N. Machine learning techniques for ophthalmic data processing: a review. IEEE J Biom Health Inform. (2020) 24:3338–50. 10.1109/JBHI.2020.3012134 32750971

[26] Pieters W. Explanation and trust: what to tell the user in security and AI? Ethics Inform Technol. (2011) 13:53–64. 10.1007/s10676-010-9253-3

[27] Wang Y-L Yang J-Y Yang J-Y Zhao X-Y Chen Y-X Yu W-H. Progress of artificial intelligence in diabetic retinopathy screening. Diabetes Metab Res Rev. (2020):e3414. 10.1002/dmrr.3414 33010796

[28] London AJ. Artificial intelligence and black-box medical decisions: accuracy versus explainability. Hastings Center Rep. (2019) 49:15–21. 10.1002/hast.973 30790315

[29] Perdomo O Rios H Rodríguez FJ Otálora S Meriaudeau F Müller H. Classification of diabetes-related retinal diseases using a deep learning approach in optical coherence tomography. Comput Methods Progr Biomed. (2019) 178:181–9. 10.1016/j.cmpb.2019.06.016 31416547

[30] Pedrosa M Silva JM Silva JF Matos S Costa C. SCREEN-DR: collaborative platform for diabetic retinopathy. Int J Med Inform. (2018) 120:137–46. 10.1016/j.ijmedinf.2018.10.005 30409338

[31] Sherlock A Brownie S. Patients' recollection and understanding of informed consent: a literature review. ANZ J Surg. (2014) 84:207–10. 10.1111/ans.12555 24812707

[32] Glaser J Nouri S Fernandez A Sudore RL Schillinger D Klein-Fedyshin M. Interventions to improve patient comprehension in informed consent for medical and surgical procedures: an updated systematic review. Med Decis Mak. (2020) 40:119–43. 10.1177/0272989X19896348 31948345 PMC7079202

[33] Balthazar P Harri P Prater A Safdar NM. Protecting your patients' interests in the era of big data, artificial intelligence, and predictive analytics. J Am Coll Radiol. (2018) 15(3 Pt B):580–6. 10.1016/j.jacr.2017.11.035 29402532

[34] Martinez-Martin N Kreitmair K. Ethical issues for direct-to-consumer digital psychotherapy apps: addressing accountability, data protection, and consent. JMIR Mental Health. (2018) 5:e32. 10.2196/mental.9423 29685865 PMC5938696

[35] Morley J Machado CCV Burr C Cowls J Joshid I Taddeo M. The ethics of AI in health care: a mapping review. Soc Sci Med. (2020) 260:113172. 10.1016/j.socscimed.2020.113172 32702587

